# Childhood osteomyelitis: imaging characteristics

**DOI:** 10.1007/s13244-012-0186-8

**Published:** 2012-08-10

**Authors:** Joost van Schuppen, Martine M. A. C. van Doorn, Rick R. van Rijn

**Affiliations:** 1 Department of Radiology, Academic Medical Center/Emma Children’s Hospital, Amsterdam, The Netherlands; 2 Department of Radiology, Academic Medical Center/Emma Children’s Hospital, Meibergdreef 9, 1105 AZ Amsterdam Zuid-Oost, The Netherlands

**Keywords:** Paediatrics, Musculoskeletal diseases, Osteomyelitis, Radiology, Diagnostic imaging

## Abstract

**Background:**

The purpose of this review is to illustrate the imaging findings of childhood osteomyelitis. The diagnosis of childhood osteomyelitis can be challenging. Clinical presentation and laboratory results can differ and are relatively unreliable. To date, its role in the assessment of treatment efficacy is not yet clear.

**Methods:**

This review article provides an overview of the different imaging modalities and imaging characteristics of childhood osteomyelitis. Levels of evidence for different modalities are presented.

**Results:**

Paediatric radiology plays a pivotal role in the diagnosis of childhood osteomyelitis and can also be used to guide therapy and intervention.

**Conclusion:**

Although imaging is essential in the diagnostic process, cooperation between the physician and radiologist remains the cornerstone in accurately diagnosing childhood osteomyelitis.

**Main Messages:**

• *Imaging plays a pivotal role in the diagnosis of childhood osteomyelitis.*

• *Cooperation between the clinician and radiologist is a very important aspect of making the diagnosis.*

• *The initial imaging modality in childhood osteomyelitis is conventional imaging.*

• *Normal conventional imaging does not exclude osteomyelitis.*

## Introduction

Childhood osteomyelitis can be a challenging problem to both the clinician and the radiologist as the clinical presentation can be variable in severity, and laboratory results are relatively unsupportive in the diagnostic workup. Radiology plays a pivotal role in its diagnosis and can also be used to guide therapy and intervention. This review discusses the pathophysiology of childhood osteomyelitis and the clinical presentation. With respect to radiological imaging, it will focus on the application of radiological techniques in the diagnosis of childhood osteomyelitis and the reported levels of evidence for the use of these techniques (Table 1).

**Table 1:** Classification of levels of evidence used in this review [18]

Level	Description
Level I	Strong evidence based on studies with a broad generalisability or meta-analyses based on level I studies
Level II	Moderate evidence based on prospective or retrospective studies with narrow generalisability or cohort studies, case control studies or randomised control trials
Level III	Limited evidence based on diagnostic accuracy studies with several flaws in research methods or on nonrandomised comparison studies based on outcomes
Level IV	Insufficient evidence, studies with multiple flaws in research methods or case series, descriptive studies or expert opinions

## Osteomyelitis

### Pathophysiology

Osteomyelitis is defined as an infection of the bone, bone marrow and surrounding soft tissue. In childhood the most common route of infection is haematogenous spread of a microorganism. Secondary spread by contiguity and direct spread of infection, for example due to direct penetration e.g. in cases of comminuted fractures, are seen less often [2, 3]. Osteomyelitis can also follow after trauma, where a metaphysical haematoma can function as a focus of infection [4]. Osteomyelitis as a result of vascular insufficiency, e.g. as commonly seen in adult diabetic patients, is seldom seen in childhood and will therefore not be discussed in this review.

In order to understand the clinical and radiological findings in childhood osteomyelitis, it is important to recall the anatomy of the paediatric skeleton. In growing bone the diaphysis and metaphysis share the same nutrient arteries and veins. These arteries and veins form a fine network of arterioles and venules in the metaphysis, which leads to the formation of so-called sinusoidal lakes. These sinusoidal lakes can act as pools where microorganisms can accumulate, thus leading to a focus of osteomyelitis. In contrast to the adult situation, in young children the epiphysis has its own nutrient vessels. After 12 to 18 months of age, these transphysial vessels disappear. As a result the physis acts as a natural border and prevents the spread of osteomyelitis from the metaphysis to the epiphysis. Spread to the epiphysis and joints is less common.

Neonates are more prone to osteomyelitis. Because of a less developed immune system, osteomyelitis can be caused by less virulent agents and tends to present fewer clinical signs. The combination of unclear symptoms in neonates and the presence of transphysial vessels can lead to indolent infections that are often discovered at a late stage [3, 4].

The reported annual incidence of childhood osteomyelitis is 3 to 20 per 100,000. For acute osteomyelitis the incidence is 8 per 100,000 and for sub-acute osteomyelitis 5 per 100,000 [2, 3, 5, 6]. The incidence is higher in children below 3 years of age, with a peak incidence in children below 1 year of age. Acute osteomyelitis occurs more often in boys, with a reported male-female ratio of 1.9 to 1.0 [2, 3, 5–7].

The most common site of infection is the long bones, especially the femur and tibia. Most infections are mono-ostotic, but polyostotic involvement of up to 6.8 % is reported in infants, and even 22 % in neonates [6].

Risk factors for osteomyelitis include trauma, sickle cell disease, immunodeficiency, sepsis, minor trauma in combination with bacteraemia, an indwelling vascular catheter and chronic vascular lines, for example in case of haemodialysis [4, 7].

The differential diagnoses based on radiological features for acute and chronic osteomyelitis are summarised in Table 2.

**Table 2:** Differential diagnosis of acute and chronic osteomyelitis in children

Differential diagnosis	Major imaging or clinical feature
Acute osteomyelitis	
Vaso-occlusive disease	Linear hypointense on T1- and T2-weighted changes in meta- and epiphysis
Septic emboli	Growth plate involvement in fulminant meningococcemia
Septic arthritis	Fluid in joints
Spondylodiscitis	Imaging shows low signal of the disc with fluid/abscess around it with destruction of the vertebrae, rim enhancement after gadolinium. (image 9)
Osteoid osteoma	Cortical sclerotic lesion with typical lucent nidus
ALL	Diffuse bone marrow changes, T1 low signal and T2 heterogeneous
Stress fracture	Linear lesions show hypointense changes on T1, without enhancement
Metastastic neuroblastoma	Multiple lesions with high signal on STIR. In context of neuroblastoma
Ewing’s sarcoma	Large soft tissue mass, onion-skin periostitis, metastasis
Osteosarcoma	Codman’s triangle, sunburst spiculated periostitis, cortical destruction
Self-limiting sternal tumours of childhood (SELSTOC)	Ultrasound shows dumbbell-shaped lesions extending to the area behind the sternal bone, involving the cartilage, leading to increased distance between ossification centres
Chronic osteomyelitis	
Ewing’s sarcoma	Large soft tissue mass, onion-skin periostitis, metastasis
LCH	Typical punched-out lesion on conventional imaging. Whole-body MRI STIR can be used for screening
Metastasis	Multifocal lesions, no inflammation parameters
CRMO	STIR and T2 series show multiple spots of high signal intensity, and series after contrast show enhancement. Imaging characteristics are comparable with acute osteomyelitis. Focus of osteomyelitis and symptoms can change over time. PET scan can also show multiple sites of uptake

### Clinical findings and laboratory tests

Clinical presentation can be diverse and therefore confusing. Usually pain and reluctance to move limbs are present. Also fever, swelling and tenderness can be present. Acute haematogenous osteomyelitis (AHOM) is defined as the presence of complaints for fewer than 14 days, whereas the sub-acute form persists longer than 14 days.

In osteomyelitis with anaerobic organisms, even the presentation can be without symptoms or just mild systemic symptoms such as low fever can be present [8].

A study by Riise et al. showed that an erythrocyte sedimentation rate (ESR) of more than 40 mm/h has the highest predictive value (26 %) [6]. All other tests had a lower predictive value. Blood cultures were only positive in 26 % of cases of acute osteomyelitis and negative in 100 % of cases of sub-acute osteomyelitis. Other authors report a higher percentage of positive blood tests [9]. The white blood cell count can be normal, whereas C-reactive protein (CRP) and ESR levels are raised in most cases [6].

The most common organism causing acute haematogenic osteomyelitis is Staphylococcus aureus (up to 95 %), followed by β-haemolytic Streptococcus, Streptococcus pneumoniae, Escheria coli and Pseudomonas aeruginosa. Besides these microorganisms, fungi, viruses and parasites have been reported to cause childhood osteomyelitis (Table 3) [3, 9].

**Table 3:** Causative pathogens in childhood osteomyelitis [10, 16, 24, 26]

Causative pathogens	Incidence
Staphylococcus aureus/MRSA	30–95 %
Streptococcus pneumoniae	0.5–17 %
Streptococcus pyogenes	17 %
β-Haemolytic streptococcus	0.5–6 %
Pseudomonas aeruginosa	4.2 %
Group A Streptococcus bacterium	4.2 %
Kingella kingae	1.4 %
Escheria coli	0–0.5 %
Candida albicans/Coccidioides immitis	0.5 %
Aspergillus	Unknown
Tuberculosis^a^	Unknown
Salmonella	In case of sickle cell disease
Parasites^a^	Unknown
Anaerobic	Unknown[8]
Unknown (no positive culture)	25–38 %

^a^Seen in endemic areas

In recent years the prevalence of methycillin-resistant Staphylococcus areus (MRSA) osteomyelitis has increased. MRSA leads to more aggressive cases of osteomyelitis, also with a higher prevalence of abscess formation and other complications such as myositis and pyomyositis [11].

Specific pathogens strongly depend on age, the immune status of the patient and the geographic location for endemic diseases, such as tuberculosis [12].

Since the introduction of the Haemophilus influenza type b vaccination in the national vaccination programs, the incidence of septic osteomyelitis and arthritis due to this organism has been significantly reduced. Nevertheless, this organism should be kept in mind as a causative agent in areas without a vaccination programme and also in possible cases of failure of the vaccine [8, 13].

In sickle cell disease the diagnosis of osteomyelitis can be more challenging, and in these patients clinical signs and laboratory testing can be misleading. Pain in joints and bones can be a sign of a sickle cell crisis/osteonecrosis but also of osteomyelitis. Interpretation of imaging, especially MR, as discussed below, can be challenging when differentiating between osteomyelitis and sickle cell crisis/osteonecrosis [14].

### Complications and outcome

A complication of osteomyelitis can be the development of an abscess, typically known as Brodie’s abscess. Also the development of sequesters, fistulas and sinus tract lesions can be seen. Although CT and MR imaging is useful in delineating the extension of abnormalities, it is difficult to differentiate between an active or an inactive focus of osteomyelitis. In these cases, PET-CT, which has a high sensitivity for disease activity, might play a role [15]. Other reported complications are septic arthritis, slipped epiphysis, damage of the physis causing early closing of the physis and eventually leading to growth retardation, or angulation deformation in the long bones [16].

### Principles of treatment

Ideally treatment is based on the isolation of the pathogen from the focus of infection or blood. If this is not possible, empirical treatment, consisting of intravenous antibiotics in haematogenous osteomyelitis, should be started. Once the pathogen is known, treatment should be changed if needed. This approach has been shown to be effective, resulting in a cure rate of over 95 % [17]. In a systematic review, no significant difference was found between a short (less than 7 days) and a long (1 week or longer) course of intravenous antimicrobial therapy when the clinical cure rate at 6 months was the primary outcome variable [18].

Surgical intervention is used as an adjunct to antibacterial treatment. The aim of surgery should be to drain intra-osseous abscesses, removal of sequestra and debridement of adjoining infectious foci. Interventional radiology plays a role in percutaneous drainage of soft-tissue abscesses. Duration of therapy depends on the effect of therapy and surgical intervention [16]. For the latest standard in osteomyelitis treatment, the reader is referred to Up-to-Date [7].

Poly-microbacterial infections give rise to more complications. Response to treatment is often monitored by ESR and CRP. Follow-up imaging may be used to evaluate complications [19].

#### Imaging techniques

##### Conventional imaging

Conventional radiography is the initial modality of choice to evaluate osseous changes. In the majority of cases it will be the only imaging technique used in the diagnosis and treatment of childhood osteomyelitis.

The ease of access and the relatively low radiation dose make it an ideal imaging tool for skeletal pathologies. Also it is possible to exclude other pathologies such as malignancies and fractures. However, conventional imaging should not be used to exclude osteomyelitis in the first 10 days of symptoms [4, 20].

Karmazyn et al. advise performing at least two orthogonal views of the body part of interest [19]. Comparison with radiographs of the opposite limb is seldom useful, and therefore they should not be routinely obtained.

Conventional imaging has a reported sensitivity of 20–75 % and a specificity of 75–83 %, but evidence is limited (level of evidence II–III) [2–4, 9].

##### Ultrasound (US)

Ultrasonography, especially in children, is a very useful and versatile modality. It allows correlating the physical exam with US findings and comparing the affected side with the opposite side [3, 21, 22]. It is important to use a linear high-frequency transducer.

**Fig. 1:**
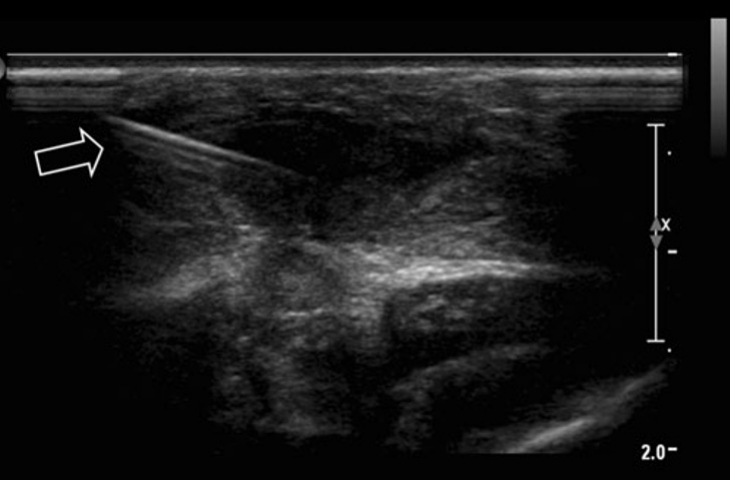
Ultrasound-guided biopsie needle aspiration of a subcutaneous pre-sternal abscess

US has a high reported sensitivity for detection of sub-periostal abscesses and fluid collections [3, 22], also complications such as soft tissue abscess, sequesters, fistulas and sinus track formation can be evaluated by US. Furthermore, it is possible to perform direct intervention, such as aspiration or drainage of an abscess or joint effusion (Fig. 1). A drawback of US is the inability to evaluate bone marrow involvement and the operator dependency. The sensitivity of US in osteomyelitis is reported to be 46–74 %, with a high specificity of 63–100 % (level of evidence: III) [21, 23].

##### Computed tomography (CT)

In the acute workup of osteomyelitis, computed imaging plays no role.

**Fig. 2:**
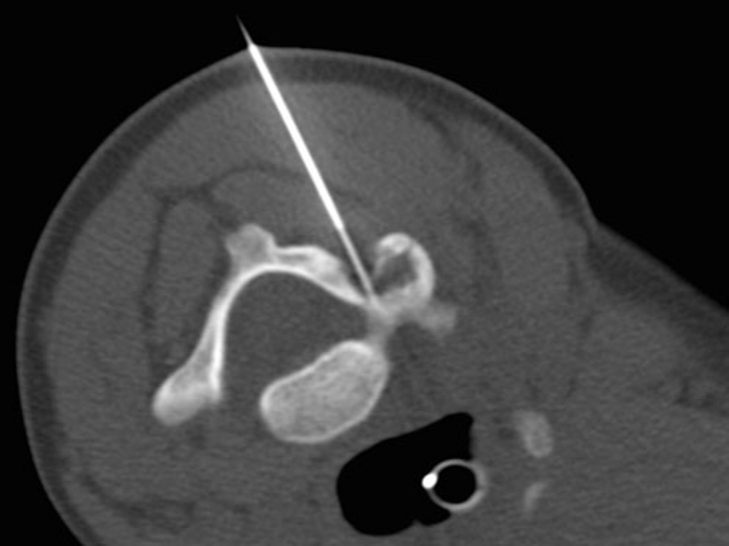
A 15-month-old boy suspected to have spondylodiscitis. CT-guided needle aspiration was performed for analysis

In chronic osteomyelitis, it is more useful and superior to MR in detecting cortical destruction, air and sequesters [3, 4, 19, 20, 24–26]. The main advantage of CT is the high spatial resolution of bony structures and surrounding soft tissue, and can also be used in the diagnostic workup of CT for image-guided aspiration/intervention (Fig. 2).

A major disadvantage of CT is the relatively high amount of radiation; therefore, CT should be used according to the ALARA principle (As Low As Reasonably Achievable) [27].

No specific literature was found on the sensitivity and specificity of CT in detecting acute osteomyelitis and its complications. Based on a systematic review, the sensitivity and specificity of CT in chronic osteomyelitis were found to be respectively 67 % and 50 % (level of evidence: III) [28].

##### Magnetic resonance imaging (MRI)

After conventional imaging MRI is the most important imaging modality for the evaluation of osteomyelitis. MRI not only allows excellent delineation of the osseous component but also soft tissue extension, joint effusion and complications of osteomyelitis such as abscess formation, especially in case of failure to respond to therapy [25].

In young children the main disadvantage of the use of MRI is the long scan time during which the patient has to remain immobile. This leads to the need for general anaesthesia (intubation) or sedation in young children. Other approaches, such as immobilisation with for example a vacuum mattress or a feed and swaddle protocol, have been advocated, thus obviating the need for general sedation [29]. For older children having enough time and patience to make the child feel comfortable and cooperative is mandatory. Adaptations to the environment, such as the use of an open MRI, the presence of parents or distraction during imaging, can also be helpful.

First of all, it’s important to choose the appropriate coil for the maximum zone of interest and adequate details. There always needs to be a balance between the signal-to-noise ratio and image resolution. In children, it is wise to start with the most important sequences, in case the examination has to be stopped before the end of the planned scan. Long sequences should be avoided, but if really needed, they can be separated in contiguous series. The most important sequences for the diagnosis and follow-up of osteomyelitis are summarised in Table 4 [30]. The use of gadolinium-enhanced MR is still under debate, especially when unenhanced series are normal. Nevertheless, research by Kan et al. shows that the sensitivity of contrast-enhanced MR is higher for abscesses than that of non-enhanced MR. Indications for using contrast are spinal osteomyelitis and cases where the initial studies are inconclusive and clinical symptoms persist [4, 31].

**Table 4:** Sequences and indications

T1 SE/FSE	Excellent sequence for illustration of anatomy, bone marrow (conversion), fat content, haemorrhage, calcifications, fracture line, tumor margins, soft tissue. It pairs a good image quality with a high spatial resolution
T2 FSE +/- FS	Sensitive for oedema, bone marrow, effusion and soft tissue (muscle oedema)
Contrast enhanced T1 FS	Fat suppression technique in combination with gadolinium, make it easier to see the enhancement
	Contrast will change sensitivity and specificity and increases the confidence in making the diagnosis of osteomyelitis on MR.
Delayed sequence (3-10 minutes)	High spatial resolution, or dynamic contrast series 3D GRE T1for functional imaging (perfusion) with high temporal resolution (3-15sec) and total acquisition time of 5 minutes
SE T1 pre/post injection (subtraction image),	
SE T1 FS	
3D GRE FS	
Dynamic series	Are good for post-treatment evaluation. You should inject contrast agent if STIR/T2 and T1 sequences are normal.
STIR	Sensitive to oedema. It gives a homogenous fat saturation and the possibility of a large field of view. STIR sequences never after gadolinium injection, because the signal of gadolinium is suppressed
PD SE/FSE (FS)	Perfect for anatomy, oedema. Also for evaluating meniscus, ligaments (SE>FSE), articular cartilage, growth cartilage (zone of provisional calcification), and bone marrow (FS)
Gradient Echo FS	Excellent for cartilage, with 3DT1 or T2* is the best sequence for blood products
DWI	Perform a b=0 for the T2-shine-trough-effect. There is not enough evidence for the use of diffusion weighted imaging in osteomyelitis, although in the evaluation of osteomyelitis treatment this could have an application.
Whole body MRI	Evaluation of metastases/LCH, can be performed in case of multifocality

MRI has a high sensitivity for detection of osteomyelitis of 82–100 % and high specificity of 75–99 % (level of evidence: III) [3, 19, 24]. In cases of multifocal sites or if the localisation of the symptoms is doubtful, a whole-body MRI can be performed, although there is poor evidence (level of evidence: IV).

##### Nuclear imaging

Bone scintigraphy can play a role when localisation of osteomyelitis, based on clinical information or other radiological methods, is not possible [32]. However, in the last few years nuclear imaging has increasingly been replaced by whole-body STIR MRI, thus allowing for one-stop-shop imaging.

The use of FDG-PET CT in the diagnosis of osteomyelitis has mainly been reported in adults [33]. For paediatric use a drawback is the high effective radiation dose, which can be in the range of 5–18 mSv [34].

Although limited evidence is available on paediatric osteomyelitis, PET CT can effectively differentiate between an active and inactive focus of chronic osteomyelitis [19]. Literature on the use of FDG PET in adults shows a sensitivity of 94–100 % and a specificity of 75–99 % (level of evidence: IV) [19].

## Imaging findings in osteomyelitis

### Strategy

**Fig. 3:**
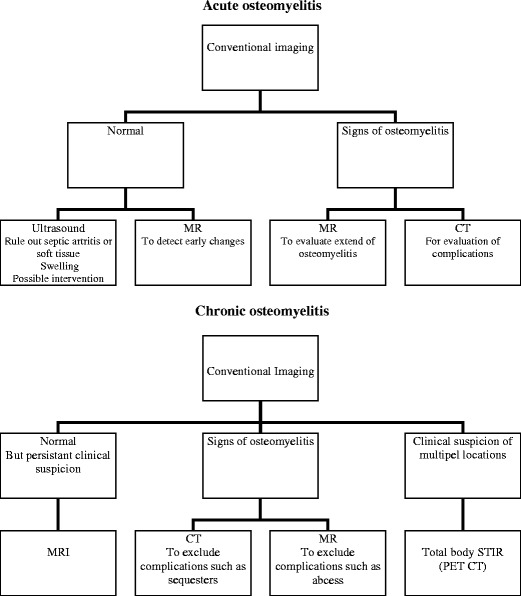
Flow chart of proposed imaging strategy

The different modalities and their advantages and disadvantages are discussed above. The imaging strategies for these modalities in the setting of acute and chronic osteomyelitis are summarised in a flowchart (Fig. 3).

## Acute osteomyelitis

Acute haematogenous osteomyelitis is typically seen in young children and is clinically characterised by a rapid onset of complaints after contact with a pyogenic blood-borne organism. Its typical localisation is the metaphysis of the tibia and femur. Through the transphyseal nutrient vessels, spreading to the physis, epiphysis and joints is possible.

In children, compared to adults, the periostium is loosely attached to the bone, and in case of infection it can easily be lifted, thereby creating a space for pus collection.

Multiple foci of osteomyelitis can be seen in children with sickle cell disease, diabetes mellitus and chronic granulomatous disease [20].

### Subacute osteomyelitis

**Fig. 4:**
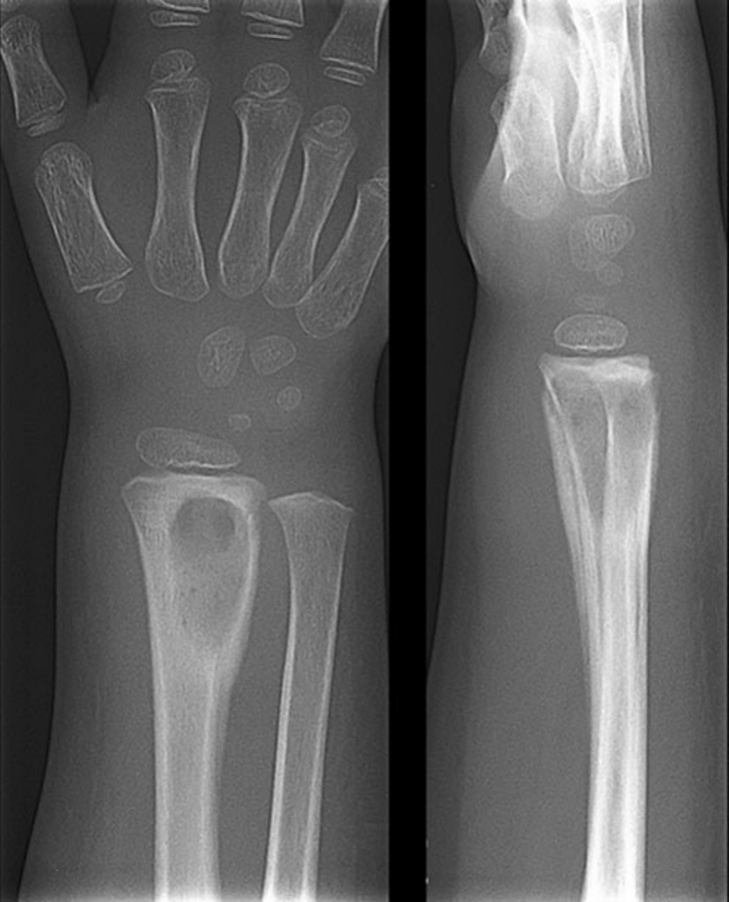
A 3-year-old boy with osteomyelitis of the distal radius. Anterior-posterior and lateral conventional radiographs show a sharply delineated lucent lesion in the distal radius

An osteomyelitis with symptoms longer than 2 weeks is defined as subacute osteomyelitis and shows more classical signs on conventional imaging, including single or laminated periostal reactions, and well-circumscribed metaphysical lucency in the long bones, also known as Brodie’s abscess (Fig. 4) [20].

#### Imaging findings

##### Conventional imaging

**Fig. 5:**
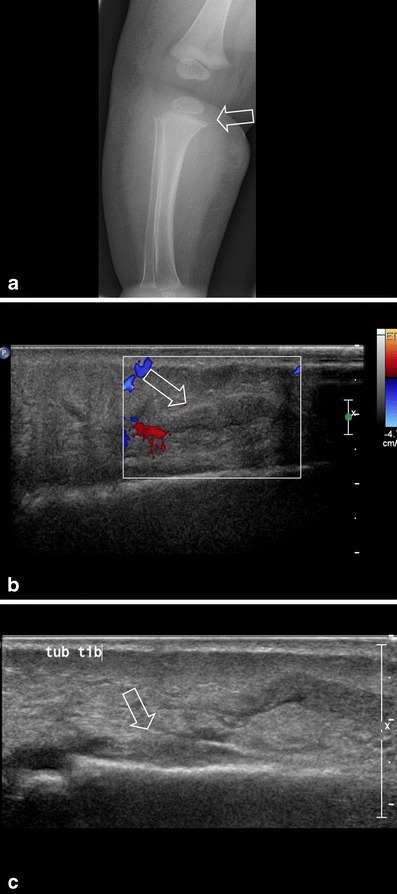
**a** A 3-month-old girl with tenderness of the right leg and fever. Imaging shows soft tissue swelling around the tibia. The periostium of the proximal metaphysis is slightly irregular (*arrow*). **b** Ultrasound with Doppler shows infiltration of the subcutis, hyperaemia and a long subcutaneous fluid collection (*arrow*). **c** Subperiostal fluid collection/abscess (*arrow* shows uplifted periostium). Surgically the abscess was drained and the patient received antibiotics

In the first 48 h, deep soft tissue swelling in the metaphyseal region and loss of fat planes can be visible on conventional imaging (Fig. 5).

**Fig. 6:**
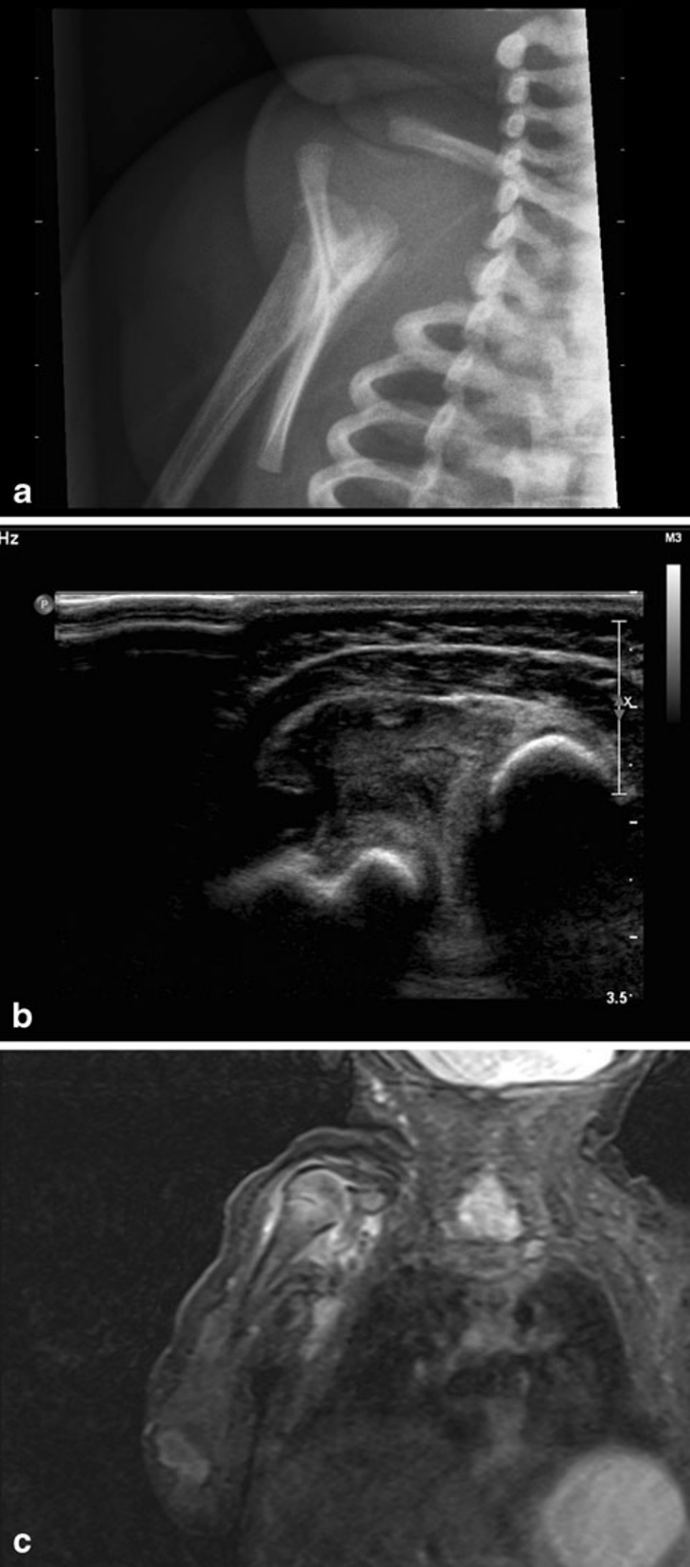
**a** A 1.5-month-old girl with low grade fever and reluctance to move her right shoulder. Conventional imaging shows a lucent lesion with possible cortical disruption dorsal in the head of the humerus and periostal reaction in the proximal third of the humerus. **b** Ultrasound study revealed hyperechoic fluid in the shoulder joint, as seen in septic arthritis. **c** Coronal T1 STIR series, although with motion artefact, showed high signal around the humeral head and high signal in the bone marrow of the proximal third of the shaft. The final diagnosis was septic arthritis in combination with osteomyelitis of the proximal humerus

**Fig. 7:**
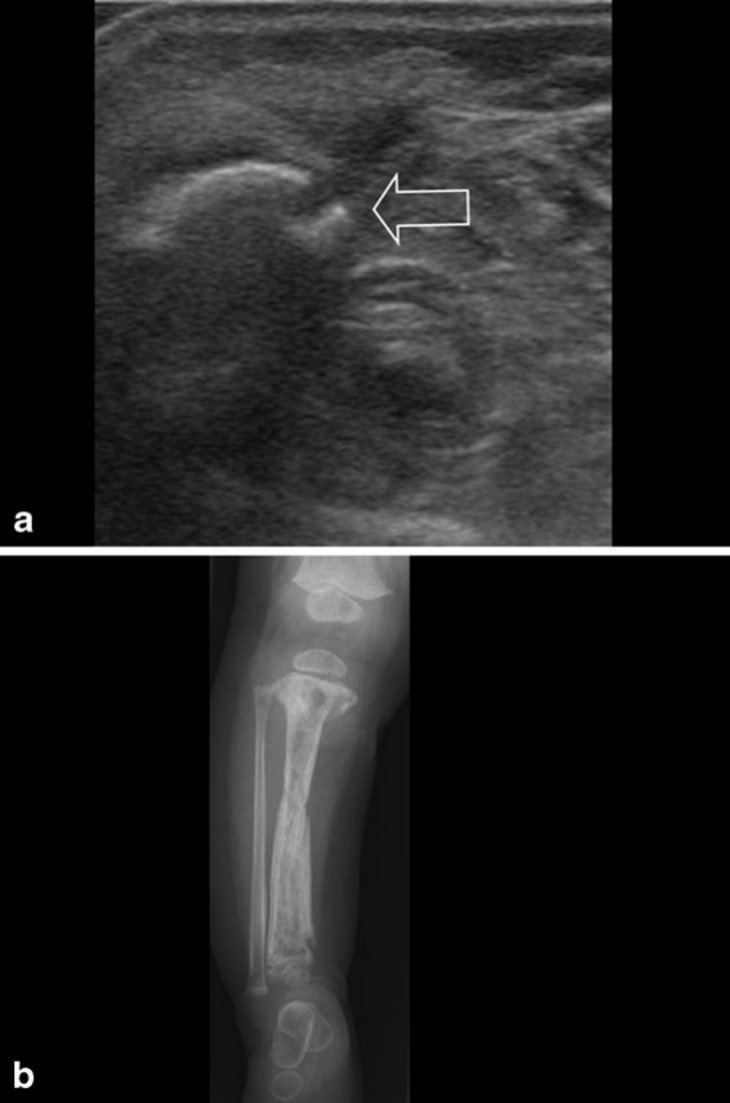
**a** Same patient as in Fig. 4 was treated with antibiotics for 1 month. Because of low-grade fever, ultrasonography was repeated. A rest of the abscess was seen as well as extensive cortical destruction at multiple locations (*arrow* shows one). **b** Conventional image was performed to evaluate the extensiveness, showing complete involvement of the tibia, with fracture lines in the proximal and distal metaphysis, and mid diaphysis

**Fig. 8:**
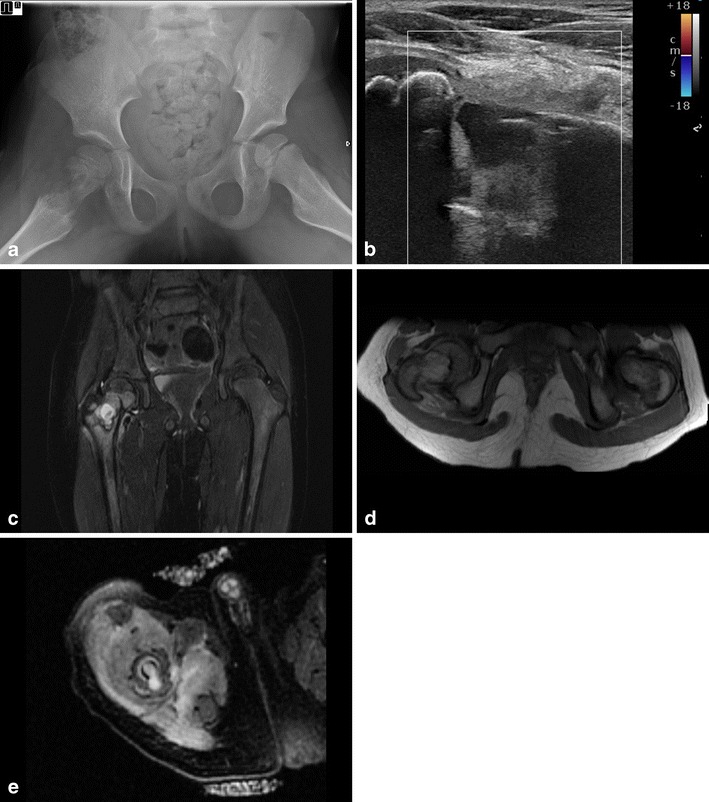
**a** A 3-month-old boy with a fever and reluctance to use his right limb. Physical examination shows a swollen, red hip. Conventional imaging shows extensive destruction of the proximal right femur and swollen soft tissue. b Ultrasound of the right hip shows cortical destruction, the formation of a subperiostal fluid collection/abscess and infiltration of soft tissue. **c**–**d** Coronal T1 STIR and axial PD images show cortical destruction and fluid collection/abscess formation in bone marrow. No contrast-enhanced imaging was performed in this case. **e** Axial T2 FS image confirms the fluid/abscess collection with extension outside the bone

Several days after the onset of symptoms, periostal reactions can be seen (Figs. 6 and 5), and after 7 to 21 days lytic lesions can be seen in affected bones (Figs. 6 and 4). Eventually, this can lead to marked destruction of the bone with extension into the cortex (“endostal scalloping”). Bone destruction can appear as lucency but also in a permeative pattern. Destruction can be limited to the metaphysis, but can also, in rare cases, if misdiagnosed or left untreated, extend into the physis and epiphysis (Fig. 7). This can be very difficult to diagnose on conventional radiographs because in children the epiphysis is not yet ossified [3, 4, 6, 19, 26]. The presence of a joint effusion can be a helpful clue, although plain radiographs have a low sensitivity for detecting joint effusion (Fig. 8).

##### Ultrasound

US allows evaluation of soft tissue swelling due to oedema or fluid collection, and hyperaemia can be detected using Doppler techniques. Also periosteal thickening and sub-periostal collections are seen. These findings can precede abnormalities on conventional imaging in the first week (Figs. 5 and 7).

US has high sensitivity for detection of intra-articular fluid [3, 4, 21, 24, 25, 30] (Figs. 6 and 8).

**Fig. 9:**
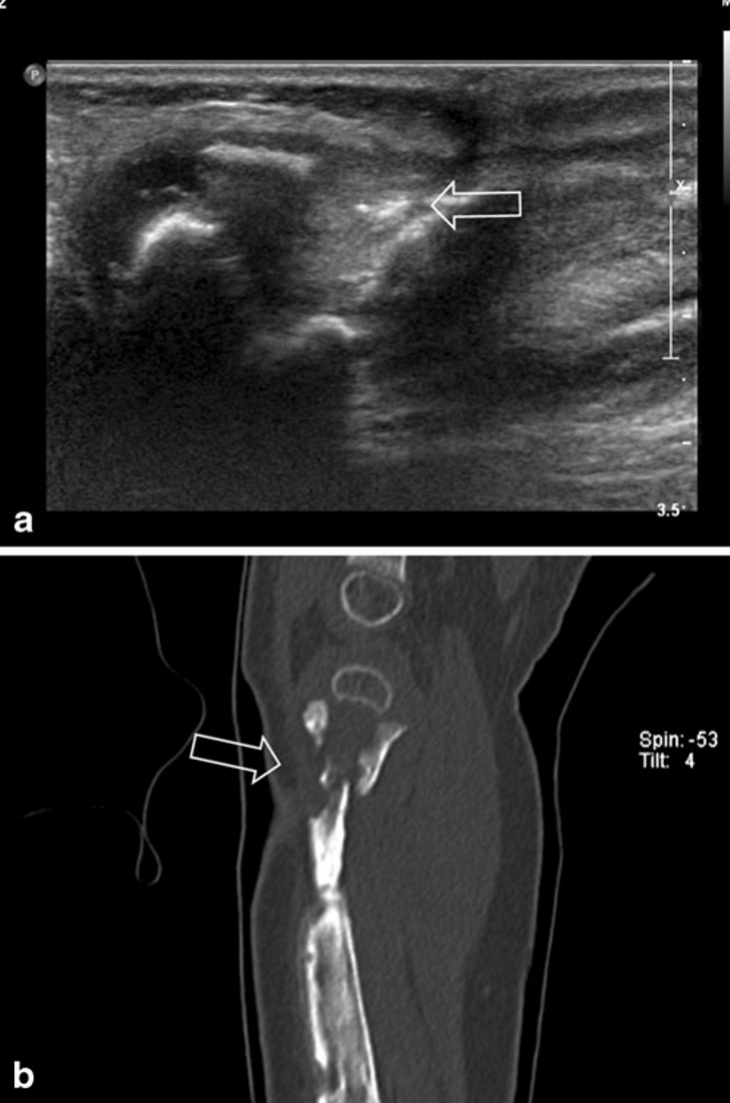
**a** A 3-month-old girl after treatment for osteomyelitis. In the follow-up, there was the suspicion of a sequester in the proximal metaphysis. Ultrasound was performed, which showed hyperechoic fragments in a surrounding fluid collection, suspicious for a small sequester (*arrow*). **b** CT confirmed the presence of a small sequester (*arrow*)

In more extensive cases, cortical defects can also be detected (Fig. 8). It can be useful for detecting foreign bodies, especially non-radioopaque foreign bodies such as wood splinters as a causative agent. Sometimes it is possible to image formation of a sequesters, especially when surrounded by pus (Fig. 9).

##### Computed tomography

**Fig. 10:**
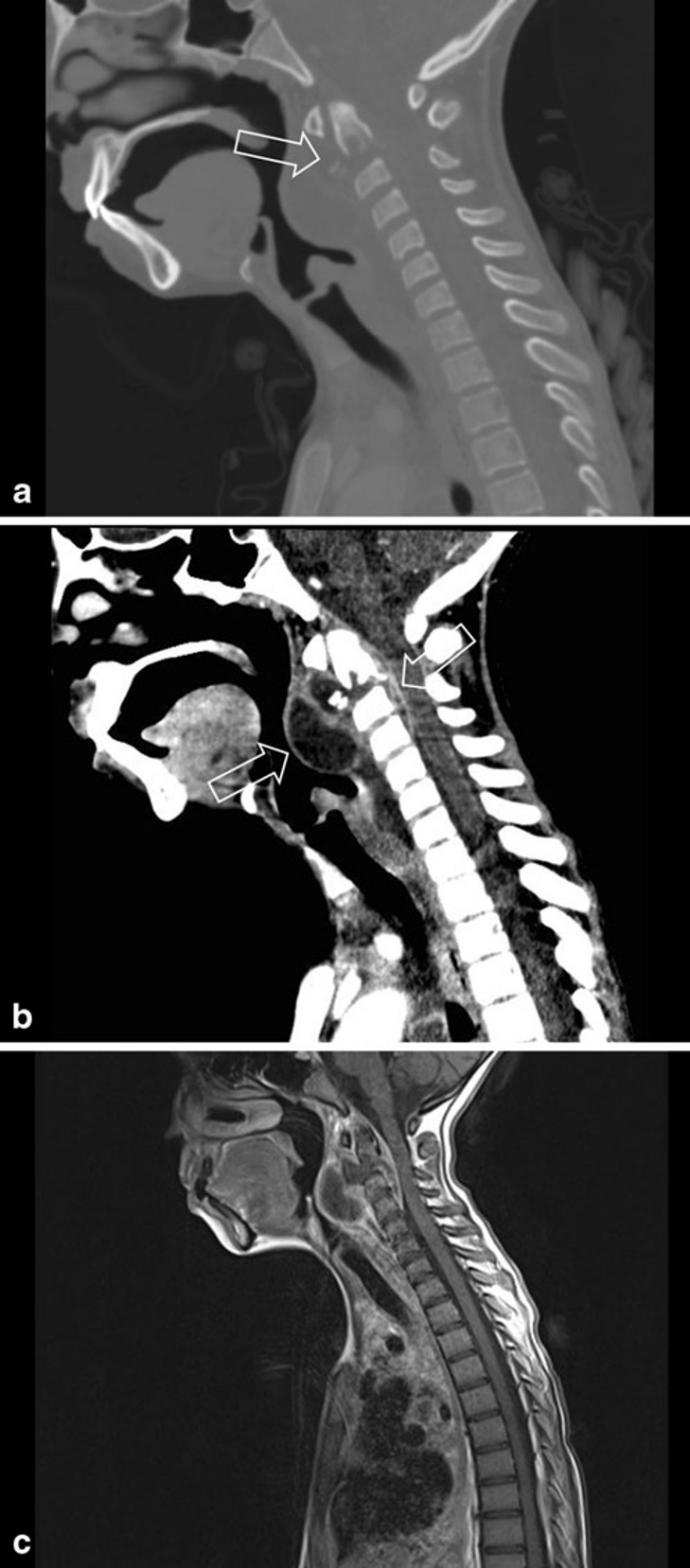
**a** A 9-year-old girl from Ethiopia, with cervical lymph nodes and weight loss, under suspicion of having tuberculosis. Fluid collection was seen on ultrasound at level II and the patient underwent CT to exclude a retropharyngeal abscess. CT showed multiple cervical and mediastinal lymph nodes, but also destruction of the end plate of the corpus of C2 with a fragment dislocated anteriorly (*arrow*). **b** CT in the soft tissue kernel clearly shows a prevertebral fluid collection with rim enhancement, but also posterior extension in the spinal canal, with enhancement. The diagnosis is a tuberculous spondylodiscitis. **c** On the sagittal T1 contrast-enhanced fat-saturated image, one can clearly see the anterior abscess, but also the intraspinal extension of the abscess in the spinal canal

CT is useful in the evaluation of bony destruction (Fig. 10) and in cases with complications such as abscess, fistula or sequester formation (Fig. 9). Intravenous contrast agents can be useful for the evaluation of soft tissue extension, although MRI has a higher sensitivity for the evaluation of soft tissue involvement (Fig. 10).

##### Magnetic resonance imaging

After 3–5 days of onset of infection, MRI can detect osteomyelitis [35]. MRI is the best technique available to evaluate changes in the bone marrow/water content of bone marrow. Alteration of bone marrow signal intensity can be visible 1 or 2 days after the onset of infection (Figs. 6 and 8) [3].

MRI criteria for diagnosis are low signal intensity on T1, high signal intensity on T2/STIR, and enhancement of (subperiosteal) bone and/or soft tissues and abscess/collections after contrast administration [36].

**Fig. 11:**
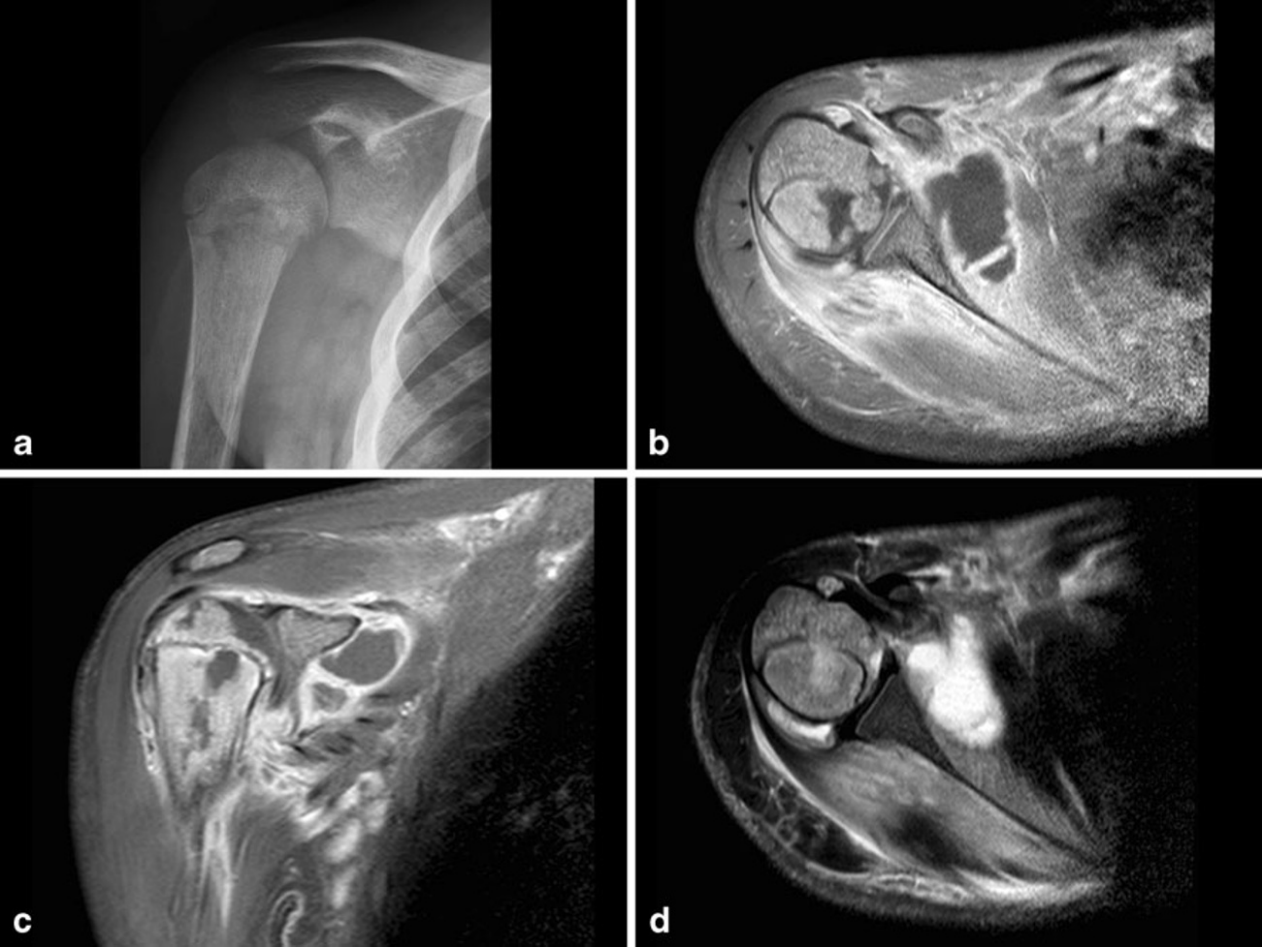
**a** A 10-year-old girl, known to have sickle cell disease, with shoulder complaints and high fever. Conventional imaging showed lucency in the humeral head and periostal reaction. **b**–**c** Axial, sagittal T1 FS contrast-enhanced series shows extensive abscess formation in the subscapular region and irregular T1 low signal intensity bone lesions. **d** Axial T2 FS shows extensive infiltration of soft tissue, abscess and also a joint effusion. This turned out to be caused by salmonella infection

MRI is also useful for evaluation of complications such as (subperiosteal) abscesses, joint effusions and soft-tissue extension that would require surgical treatment (Figs. 8, 10 and 11) [19].

##### Nuclear imaging

Acute osteomyelitis on a three-phase bone scintigraphy can show increased blood flow and blood pool activity, and positive uptake on 3 h images in areas of osteomyelitis. However, at this time blood pool flow and pool activity can be normal or photon deficient because of devascularisation of the periostium due to rapid raising of the periostium [4, 37].

PET-CT will show high uptake in areas of osteomyelitis, in bone marrow and in soft tissue.

##### Differential diagnosis (Table 2)

Since defining a differential diagnosis solely based on imaging characteristics can be very difficult, clinical and laboratory parameters and communication with clinicians remain very important in the diagnosis of osteomyelitis. In cases of doubt, fine-needle aspiration or an image-guided biopsy will eventually be necessary to determine the definitive diagnosis.

##### Patients without infectious parameters

In patients without any infectious parameters, the differential diagnosis is mainly based on disease of vascular origin, neoplasm or trauma. This group without infectious parameters can be the most challenging for the differential diagnosis.

Patients with a history of use of medications such as corticosteriods or for example patients with sickle cell disease must raise the suspicion of vaso-occlusive disease. Imaging will show typical serpentinous sclerotic lesions on conventional imaging, whereas MR imaging will show linear hypointense T1- and T2-weighted changes in the meta- and epiphysis [26].

Ewing’s sarcoma produces a large soft tissue mass without calcifications or bone matrix in the tissue. Onion-skin periostitis on conventional imaging is typical.

Osteosarcoma shows a lesion usually in the diaphysis with signs of aggressive (cortical) bone destruction and periostic reactions such as Codman’s triangle and sunburst spiculated periostitis.

In a patient with a history of trauma, stress fractures will show linear, hypointense changes on T1-weighted imaging, without enhancement. Bone marrow oedema can be visible on STIR imaging.

When imaging shows an osteolytic lesion with a central nidus surrounded by a sclerotic margin in a patient with a typical history of night pain relieved by use of NSAIDs, one should consider the diagnosis of osteoid osteoma.

In patients with suspicion of haematologic malignancy such as ALL, diffuse infiltration of the bone marrow can be seen. Imaging will show diminished signal intensities on T1 imaging; T2 will show heterogenic signals. Metastastic neuroblastoma can show multiple lesions usually characterised only by high signal intensity on STIR imaging.

In young children with a red painful or asymptomatic lesion on the sternum with or without infectious parameters, one should consider a self-limiting sternal tumour of childhood (SELSTOC) [38]. Ultrasound in these patients will show a dumbbell-shaped lesion extending to the area behind the sternal bone. No additional imaging will be needed in these patients.

##### Patients with or after a period with infectious parameters

In patients with infectious parameters in combination with backache, one must consider a spondylodiscitis. Imaging shows low signal of the disc with surrounding fluid/abscess with destruction of the vertebrae and rim enhancement after gadolinium administration (Fig. 10); Pott’s disease in tuberculosis will be more pronounced in the facet joints.

**Fig. 12:**
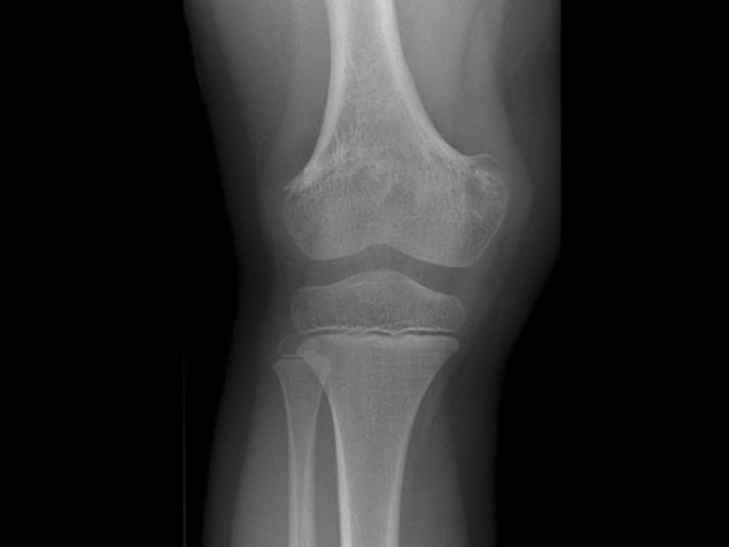
Premature closure of the epiphysis of the right femur after osteomyelitis

After a period of septicaemia, such as a fulminant meningococcemia, septic emboli can occur. This can lead to partial premature closure of the physis leading to joint deformities (Fig. 12).

#### Chronic osteomyelitis

Chronic osteomyelitis is defined as a persistent or recurrent low-grade bone infection. Usually this occurs in older patients after trauma or surgery.

##### Conventional imaging

Conventional imaging will show the same features as in acute osteomyelitis, although more extensively (Fig. 7). Complications such as fractures or sequester formation can be seen.

##### Computed tomography

CT plays an important role in the diagnosis and analysis of chronic osteomyelitis. Imaging of complications of osteomyelitis such intraosseous abscesses, sequesters, and fistulas or cloaca formation is important (Figs. 8 and 9).

Soft tissue extension can be evaluated, but to a lesser extent compared to MR. Imaging characteristics are comparable to those for acute osteomyelitis.

##### Magnetic resonance imaging

MR can show soft tissue extension and abscess. Also intraosseous abscesses and fistulas can be seen. Imaging characteristics are comparable to those of acute osteomyelitis. Use of total body STIR can exclude multifocal disease.

##### PET

PET scans will show uptake in the area of osteomyelitis. It can exclude multifocality.

##### Differential diagnosis (Table 2)

The main differential diagnosis of chronic osteomyelitis consists of neoplasms.

In Ewing’s sarcoma a large soft tissue mass without calcifications or bone matrix in the tissue can be seen. Onion-skin periostitis is typical.

LCH can be difficult to differentiate from multifocal osteomyelitis. LCH typically produces punched-out lesions on conventional imaging. Therefore, conventional imaging can provide a valuable clue.

Bone metastases are usually multifocal. In this diagnosis also the clinical parameters are important signs; in most cases, there are no signs of inflammation.

**Fig. 13:**
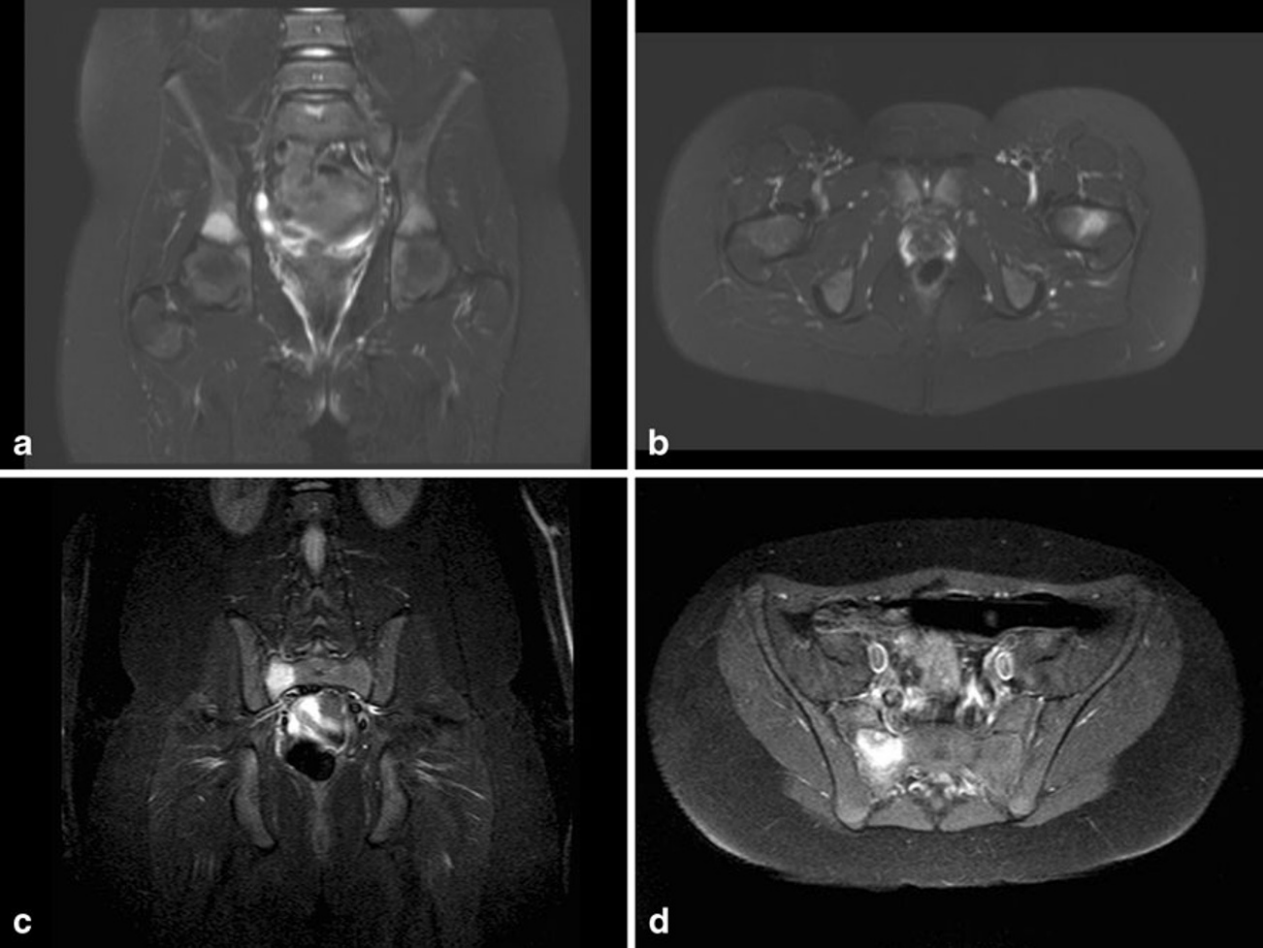
**a**–**b** An 11-year-old girl with pain in her loin. Conventional imaging did not show any abnormalities. T2 SPIR imaging showed multiple areas of high signal in both the acetabulum and the neck of the left femur. No contrast was given. Diagnosis was CRMO. **c**–**d** STIR TSE image obtained 2 months later shows only high signal intensity in the os sacrum on the right, without joint effusion of the sacroiliac joint. No other lesions were seen. T1 FS TSE contrast-enhanced series showed enhancement of the lesion

Chronic recurrent multifocal osteomyelitis (CRMO) can be part of SAPHO syndrome (synovitis, acne, pustulosis, hyperostosis, osteitis). It is a very uncommon condition, of unknown aetiology, with a peak incidence between the ages of 4–14 years. Characteristically, there are multiple locations, but only one is symptomatic [39]. The imaging strategy usually has a symptomatic focus, as in acute osteomyelitis, but multiple lesions will be shown. Especially MR and PET can be used to analyse multiple lesions. STIR and T2 series show multiple spots of high signal intensity and series after contrast show enhancement. Imaging characteristics are comparable to those of acute osteomyelitis. The focus of osteomyelitis and symptoms can change over time (Fig. 13).

## Follow-up

To date no data have been published on the use of follow-up imaging in childhood osteomyelitis. In a retrospective study by Kowalski et al. on the use of follow-up MRI for the prognosis of spinal osteomyelitis, they found that the most valid parameter on follow-up MRI was a change in soft tissue involvement. The authors concluded that only in selected cases were MR findings of additional value [40].

Only data of small-group studies on follow-up of osteomyelitis with bone scintigraphy and FDG-PET are available. These studies suggest that in the follow-up of osteomyelitis, FDG-PET and bone scintigraphy can be useful to analyse the activity of osteomyelitis and that FDG-PET is superior to MRI. Activity can be a parameter to determine the time for termination of therapy [19, 41].

**Fig. 14:**
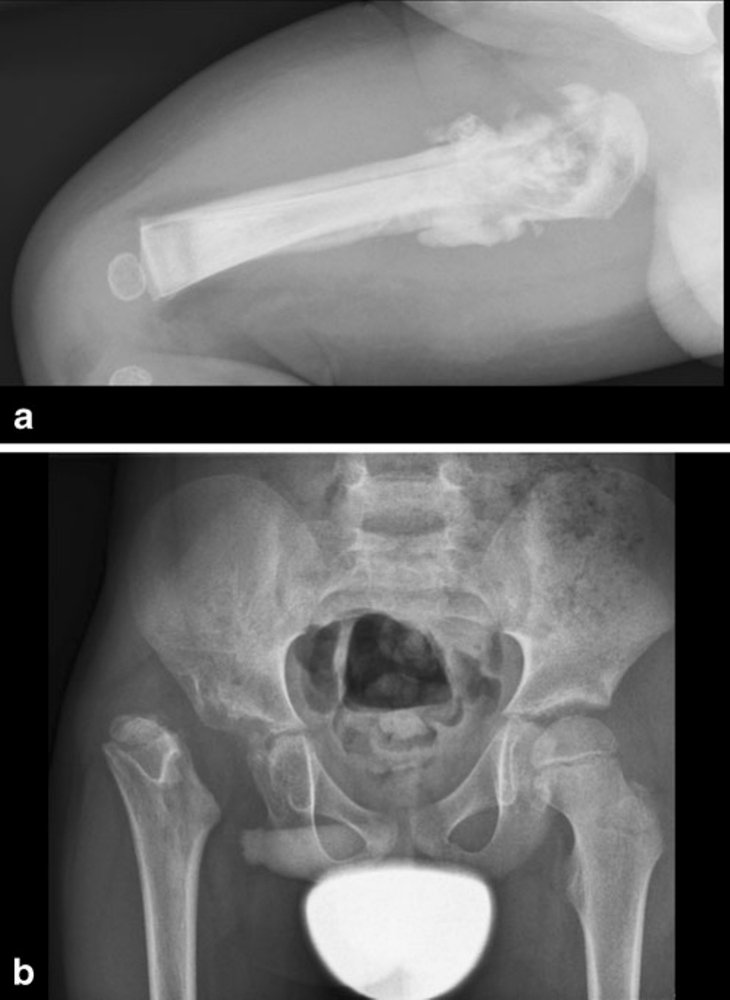
**a** A 6-week-old boy with fever. X-ray shows extensive cortical destruction of the proximal metaphysis with surrounding soft tissue calcifications and periostal reaction. This was proven to be septic osteoarthritis. **b** At age 7 years X-ray shows (acquired) deformity of the caput femoris, with luxation

Unique to childhood are the late complications of osteomyelitis due to the growth and developmental disturbances of the affected bone. These developmental changes can involve malformations of the joints (Figs. 12 and 14), leading to e.g. contractures or loss of functionality, or whole segments of bone, e.g. non-development as a result of osteonecrosis. These possible serious long-term consequences of childhood osteomyelitis indicate that these children should undergo clinical and radiological follow-up at some point in time.

## Conclusion

Osteomyelitis in childhood can be a challenging diagnosis. Imaging plays a vital role in the diagnosis of childhood osteomyelitis, and the imaging findings are pivotal in the treatment decision. As in all clinical cases the most important aspect of the diagnosis of childhood osteomyelitis lies in good cooperation among the paediatrician, paediatric orthopaedic surgeon and paediatric radiologist.

## Abbreviations

AHOM: Acute haematogenous osteomyelitis
ALARA: As low as reasonably achievable
COM: Chronic osteomyelitis
CRMO: Chronic recurrent multifocal osteomyelitis
CRP: C-reactive protein
ESR: Erythrocyte sedimentation rate
eGFR: Estimated glomerular filtration rate (ml/min)
FDG-PET: Fluorodeoxyglucose (18F) positron emission tomography
FS: Fat saturation
MRI: Magnetic resonance imaging
MRSA: Methycillin-resistant Staphylococcus areus
NSF: Nephrogenic systemic fibrosis
PD: Proton density
SAPHO syndrome: Synovitis, acne, pustulosis, hyperostosis, osteitis syndrome
SPIR: Spectral presaturation inversion recovery
STIR: Short T1/*tau* inversion recovery
TSE: Turbo spin echo
US: Ultrasound/ultrasonography
